# Airborne injustice: a preliminary exploration of the associations between pollutants and hospitalizations, sleep, and cognition in children and young adults living with sickle cell disease

**DOI:** 10.1093/jpepsy/jsaf031

**Published:** 2025-08-07

**Authors:** Shifa Hamdule, Anna M Hood, Fenella J Kirkham

**Affiliations:** Developmental Neurosciences Unit and Biomedical Research Centre, UCL Great Ormond Street Institute of Child Health, London, United Kingdom; Manchester Centre for Health Psychology, Division of Psychology and Mental Health, University of Manchester, Manchester, United Kingdom; Developmental Neurosciences Unit and Biomedical Research Centre, UCL Great Ormond Street Institute of Child Health, London, United Kingdom; Clinical and Experimental Sciences, University of Southampton, Southampton, United Kingdom

**Keywords:** sickle cell, hospitalizations, pollution, executive function, sleep, acute chest syndrome

## Abstract

**Objective:**

This study aimed to investigate the role of environmental pollutants, specifically nitrogen dioxide (NO_2_) and particulate matter (PM_10_), on children and young adults (CYA) living with sickle cell disease (SCD) in the United Kingdom. Given the heightened vulnerability of this population due to socio-environmental factors, we explored how these pollutants influence hospitalization rates, sleep quality, and cognitive function.

**Methods:**

Data were analyzed from the London Sleep Asthma Cohort, which included 94 CYA living with SCD at Visit 1, although this full sample was not available for all analyses. Participants’ exposure to NO_2_ and PM_10_ was determined using air quality data linked to their residential postcodes. Hospitalizations, sleep quality, and cognitive function were assessed through medical records, caregiver questionnaires, and cognitive testing. Multiple regression analyses were conducted to determine the relationship between pollutant exposure and health outcomes, controlling for age, community deprivation, and asthma diagnosis.

**Results:**

The study found that NO_2_ exposure significantly predicted lifetime hospitalizations for acute chest syndrome (ACS), particularly among participants with asthma. However, despite some trends toward significance, no significant relationships were observed between pollutant exposure and pain-related hospitalizations, sleep quality, or cognitive function.

**Conclusions:**

Our preliminary findings suggest that NO_2_ exposure exacerbates respiratory complications in CYA with SCD, especially in those with asthma. Our results underscore the need for targeted public health interventions to mitigate air pollution in marginalized communities, which could reduce ACS-related hospitalizations and improve health outcomes for vulnerable populations. Further research is recommended to explore the mechanisms linking pollution to SCD complications.

Environmental pollution poses a significant public health challenge, particularly in densely populated cities where concentrations of nitrogen dioxide (NO_2_; a gas primarily produced during the combustion of fossil fuels) and particulate matter (PM; everything in the air that is not a gas) are often the highest (World Health Organization, 2016). Brief exposure to NO_2_ levels exceeding 200 µg/m^3^ can lead to airway inflammation and potentially heighten the risk of respiratory infections. PM consists of a wide range of chemical substances and materials, some of which are harmful. Because many of these particles are extremely small, certain toxic compounds can enter the bloodstream and circulate throughout the body, reaching vital organs like the heart and brain. As a result, exposure to PM can have significant health consequences and has been linked to respiratory issues (such as asthma), cardiovascular disease, and lung cancer (World Health Organization, 2021). These pollutants are primarily produced by vehicle emissions, industrial activities, and residential heating, contributing to poor air quality that disproportionately affects marginalized communities (Fairburn et al., 2019).

In the United Kingdom, Black children are especially vulnerable to the harmful effects of air pollution due to the intersections of socioeconomic disadvantage, residential segregation, and systemic inequities (Al Ahad et al., 2022). Environmental exposure is of particular concern for children and young adults (CYA) living with sickle cell disease (SCD), the most common genetic disorder worldwide (Thomson et al., 2023), which predominantly affects people of African and Caribbean descent (Piel & Williams, 2016).

The interaction between environmental pollutants and the pathophysiology of SCD can lead to exacerbated health outcomes. SCD is characterized by the production of abnormally shaped red blood cells that can obstruct blood flow, reduce oxygen delivery to tissues (Rees et al., 2010), and result in heightened baseline inflammation. Although pinpointing the precise processes that initiate the chronic inflammatory state characteristic of SCD is challenging, the primary pathophysiological alteration of red blood cells has widespread effects. These include the generation of inflammatory molecules and responses that ultimately result in the blockage of microvessels, the hallmark of SCD (Conran & Belcher, 2018). Vaso-occlusion leads to the acute pain episodes (Serjeant, 2013) responsible for most hospitalizations in people living with SCD (Piel et al., 2017); these crises or pain episodes can significantly impair the quality of life of people living with SCD (Hood et al., 2021; Stokoe et al., 2022). Beyond pain, inflammation also plays a crucial role in various disease complications, such as acute chest syndrome (ACS; a severe lung-related complication) and asthma exacerbations (Khan et al., 2023). Environmental factors, such as exposure to NO_2_ and PM, could exacerbate inflammatory SCD processes by inducing systemic inflammation and oxidative stress (Aboderin et al., 2023).

Previous research has consistently demonstrated that exposure to air pollution, particularly NO_2_ and PM, is associated with adverse respiratory outcomes in children, including the development and exacerbation of asthma (Liu et al., 2021; Paciência et al., 2022). Asthma is more prevalent among children with SCD, where it often presents with increased severity (Indolfi et al., 2024). Overlapping inflammatory pathways contribute to the comorbidity of asthma with SCD, although SCD also exhibits a distinct elevated level of inflammation (Sikdar & Greenough, 2023). Asthma is associated with an increased incidence of ACS, a leading cause of morbidity and mortality in SCD (DeBaun & Strunk, 2016). ACS is characterized by fever, respiratory distress, and new lung infiltrates, often requiring hospitalization and intensive care (Jain et al., 2017). Thus, the exacerbation of asthma symptoms due to air pollution may increase the risk of ACS, leading to more frequent hospitalizations and prolonged recovery times (Field & DeBaun, 2009).

Sleep disturbances are a common and underrecognized complication in children with SCD, with poor sleep quality contributing to increased pain perception, fatigue, and reduced quality of life (Valrie et al., 2018). Exposure to air pollution has been linked to sleep disorders in children without SCD, potentially due to the irritant effects of pollutants on the respiratory system, leading to conditions such as sleep-disordered breathing (Billings et al., 2021). For children living with SCD, who are already at risk for sleep disruptions due to nocturnal hypoxemia and pain (Hollocks et al., 2012), the additional burden of air pollution may further compromise sleep quality.

In addition to sleep complications, air pollution has been implicated in children’s cognitive development, with evidence suggesting that exposure to fine PM may lead to cognitive deficits (Calderón-Garcidueñas et al., 2011). Fine PM can penetrate the blood–brain barrier, causing neuroinflammation and oxidative stress, which are thought to contribute to neurodegenerative processes (Block & Calderón-Garcidueñas, 2009). Children with SCD are already at increased risk for cognitive difficulties (Prussien et al., 2019), particularly executive function (Hood et al., 2019) and processing speed (Stotesbury et al., 2018). It is possible that the additional burden of air pollution may further exacerbate these cognitive challenges.

Some studies have examined the effects of air quality and meteorological conditions on hospital admissions in CYA living with SCD in the United Kingdom. One study found that meteorological, rather than air pollution levels near the hospital, were more likely to predict hospital admissions (e.g., pain, ACS) on rainy and windy days (Piel et al., 2017). Further research found increased hospital admissions in adults living with SCD on days when ozone (O_3_) levels were high and, conversely, when carbon monoxide and nitric oxide levels were lower in London (Yallop et al., 2006). Both of these previous studies assessed air pollution near the hospital and not near the patient’s home environment.

The compounded effects of environmental exposure to air pollutants such as NO_2_ and PM on the majority Black population of CYA living with SCD are deeply intertwined with the broader context of health disparities and environmental injustice. UK Black communities are more likely to reside in deprived communities with higher levels of air pollution due to historical and ongoing socioeconomic inequities, including limited access to green spaces and proximity to major roadways and industrial sites (Fecht et al., 2015). These inequities are perhaps starker in the United States as residential segregation, although legally abolished in the United States in 1968, remains for Black populations due to policies like mortgage discrimination and redlining (Williams et al., 2019). This segregation increases exposure to air pollutants (Woo et al., 2019). In Africa, where the SCD population is the largest, air quality is deteriorating due to increasing urbanization and poses a major health risk for people living with SCD (Cohen et al., 2017).

These environmental exposures, combined with the chronic health challenges posed by SCD, create a situation of disproportionate burden. Therefore, this study seeks to contribute to a deeper understanding of the interplay between environmental pollutants and SCD, considering the role of asthma diagnosis and community deprivation. Specifically, using longitudinal data, our preliminary study explored the multifaceted role of environmental exposure to NO_2_ and PM on children living with SCD in the United Kingdom, focusing on how these pollutants predict the frequency and severity of hospitalization rates for pain episodes and ACS, sleep quality, and cognition (i.e., executive function).

## Methods

The data underlying this article cannot be shared publicly for the privacy of the children and families who participated in the study. The data will be shared on reasonable request to the corresponding author.

### Participants

The Sleep and Asthma Cohort (SAC) study was a combined retrospective and prospective observational cohort involving children living with SCD aged 4–18 years at the time of enrollment. Recruitment occurred at three pediatric hematology centers: St Louis, Missouri, Cleveland, Ohio (United States), and London (United Kingdom). Among the 433 eligible children who were invited to participate, 283 (66%) consented, and 273 (60%) were enrolled between June 2006 and December 2009 (Visit 1), with prospective follow-up continuing until June 2013 (Visit 2) (Rosen et al., 2014). The present study represents a secondary analysis of three hospitals from the London, United Kingdom, cohort that also included a third follow-up (Visit 3) between 2016 and 2019. All enrolled patients at Visit 1 were eligible for Visits 2 and 3.

Participants were homozygous for sickle cell hemoglobin (HbSS) or compound heterozygotes for sickle B-thalassemia zero (HbSβ0) aged 4–18 years, with inclusion criteria and were enrolled without regard to past morbidity from or symptoms of sleep-disordered breathing. Exclusion criteria included receiving long-term continuous positive airway pressure therapy at the time of enrollment; participating in another clinical trial that involved blood transfusion, oxygen, or hydroxyurea therapy; a medical history of chronic lung disease other than asthma or HIV; or a personal history of smoking. Participants who had recently been placed on transfusion or hydroxyurea therapy during the study were retained in the study cohort. All participants and/or parents provided informed consent, and the study was approved by Brent (05/Q0408/42), East Midlands-Leicester (11/EM/0084), and West London (SAC; 15/LO/0347) National Health Service research ethics committees.

### Hospitalizations

A pain-related hospitalization was defined as an SCD-related pain episode requiring hospital admission and treatment with opioids. Neither Accident and Emergency (A&E) visits for pain nor hospitalizations for headaches were included in our analyses due to variations in A&E pain management and to specifically capture severe acute vaso-occlusive pain episodes, respectively. An ACS-related hospitalization was defined as one or more new radiodensities on a chest radiograph and at least one of the following: temperature greater than 38.5 °C, decreased hemoglobin oxygen saturation measured from Visit 1, or an increased respiratory rate. A hospitalization for pneumonia was considered an ACS episode. Any hospitalization in which the participant was diagnosed with both pain and ACS was classified as an ACS episode. For our analyses, we assessed pain and ACS-related hospitalizations across the lifetime (i.e., number of hospital days per year/age) and between Visits 1 and 2 (i.e., number of hospital days per year/number of years between study visits).

### Sleep quality

At Visit 2, sleep quality was assessed using an adapted version of the caregiver-report Child Sleep Habit Questionnaire (23-item CSHQ; Owens et al., 2000). The CSHQ has acceptable reliability of 0.68 and 0.78 for community and clinical samples, respectively. At Visit 2, caregivers rated their child’s sleep habits based on a frequency scale, including *never* (does not happen), *not often* (1 night/week), *sometimes* (3–5 nights/week), and *always* (6/7 nights/week). A higher total score was indicative of lower sleep quality.

### Cognitive function

CYA completed cognitive testing at Visit 3 in a quiet room. For the present study, we included the working memory index (WMI) and processing speed index (PSI) from an age-appropriate Weschler intelligence scale (WISC-IV or WAIS-4) (Wechsler, 2003, 2014). Reliability for the WISC-IV and WAIS-4 is high for the full-scale intelligence quotient (0.97 and 0.98) (0.87–94) for the specific intelligence indices. The WMI provides a CYA’s ability across subtests to attend to information presented verbally and manipulate that information in short-term, immediate memory. The PSI assesses how well an individual identifies, manipulates, and responds to information quickly.

Participants also completed the Delis-Kaplan Executive Function System (D-KEFS) Tower subtest (Delis et al., 2001), which assesses planning and problem-solving, learning rules, inhibiting impulsivity, and maintaining instructional sets. Reliability is acceptable (0.60) in clinical samples. The total achievement score is the sum of achievement points earned for all completed items and was used in analyses. A higher total achievement score represents better overall executive function.

Caregivers completed the 86-item Behavioural Rating Inventory of Executive Function (BRIEF) at baseline. The BRIEF assesses Executive Function (EF) behavior in the school and home environments (Gioia et al., 2000) and has demonstrated excellent internal consistency (0.80–0.98) and has previously been used with SCD populations (Hood et al., 2021). The BRIEF scales include Inhibit, Shift, Emotional Control, Self-Monitor, Initiate, Working Memory, Planning/Organisation, Task Monitoring, and Organisation of Materials. Our analyses used the Global Executive Composite (GEC), which incorporates scores from all scales. Higher *T*-scores (>65) indicate clinical concern (i.e., greater executive dysfunction).

### Air pollution

Air quality data were sourced from the London Air Quality Network site (London Air Quality Network, n.d.), which is a collection of urban air pollution monitoring stations in London and South East England. Each monitoring station is equipped with automated sensing devices housed in a stationary cabin, usually located near roadsides or in other high-traffic urban areas. These devices collect pollution samples approximately every 15 min, enabling short- and long-term air quality assessments. This study used the participants’ postcodes at Visit 1 to determine the monitoring station closest to their home residence. Although some participants moved between Visits 1 and 2, their subsequent residences were within the same local authority district (i.e., London borough), and they remained patients at the same hospital located in that area. We used average concentrations of NO_2_ and particles <10 µm (PM_10_). These pollutants were chosen because of their relationship to our variables of interest and because most London sites do not collect these data regularly for other pollutants (e.g., PM_2.5_ or carbon monoxide). We used two average values in our analyses. First, we averaged between Visit 1 and Visit 2 for our analyses related to hospitalizations and sleep. Next, we averaged between Visit 1 and Visit 3 for our analyses related to executive function.

### Community deprivation

The Index of Multiple Deprivation (IMD) (Department of Communities and Local Government, 2015) is a postcode-based measure of relative deprivation in England and is available as open-source government data. The IMD defines deprivation as a composite of seven domains, i.e., income, employment, education, health, crime, barriers to housing and services, and living environment. All neighborhoods are ranked on a relative rather than absolute scale according to their deprivation level relative to other areas. Areas were grouped into five bands (quintiles), with 1 = *most deprived* and 5 = *least deprived* for descriptive analyses. To increase statistical power in analyses, we grouped quintiles 1 and 2 (*most deprived*) and areas 3, 4, and 5 (*less deprived*).

### Clinical characteristics

Electronic medical records were reviewed to obtain current treatments, medical complications (i.e., history of asthma), and laboratory results (i.e., hemoglobin levels).

### Statistical analysis

We used SPSS version 28 for our statistical analyses (Rahman & Muktadir, 2021). Descriptive statistics summarized participant demographic characteristics, air pollutants, IMD, hospitalizations for pain and ACS, sleep quality, and executive function. A one-sample *t*-test determined whether the average air pollutant value across Visits 1 and 2 was above UK Air Quality Standards Regulations. The annual mean concentration of NO_2_ and PM_10_ should not exceed 40 µg/m^3^ annually. A one-sample *t*-test also determined whether participants performed below the normative mean for questionnaires related to executive function. Independent samples *t*-tests and chi-square tests examined whether demographic (e.g., age), social (e.g., deprivation), and health-related (e.g., asthma diagnosis, chronic transfusion) variables were significantly different between those with data at Visit 2 and those lost to follow-up after Visit 1.

A logistic regression analysis determined whether air pollutants predicted an asthma diagnosis for our participants. Separate multiple linear regression models investigated whether air pollutants (NOx and PM_10_) averaged across Visits 1 and 2 predicted sleep quality, hospitalizations for pain and ACS, controlling for age, IMD, and asthma diagnosis. Separate multiple linear regression models also investigated whether air pollutants averaged across Visits 1 and 3 predicted executive function, controlling for IMD. All analyses were conducted using pairwise deletion, as variables generally contained <1% of missing data. Statistical significance was determined at an α level of *p* < .05 (two-tailed).

## Results

The average time between Visits 1 and 2 and Visits 2 and 3 was 4.39 (*SD* = 0.98) years and 4.33 (*SD* = 1.62) years, respectively. There were no significant differences in age, sex assigned at birth, hospitalizations for ACS and pain, hydroxyurea use, chronic transfusion therapy, and asthma diagnosis between those with available data at Visit 2 and those lost to follow-up after Visit 1 (*p* > .05). Sociodemographic and clinical characteristic data are reported in Table 1. About half of the sample were assigned female sex at birth, almost all participants identified as Black, most lived in deprived communities, and a minority (10%) had a diagnosis of asthma and were receiving disease-modifying treatments (i.e., hydroxyurea and chronic blood transfusion).

**Table 1.** Sociodemographic and medical characteristics of participants across study visits.

Characteristics	*n*	%	*M*	*SD*
Sex assigned at birth				
Female	49	52.1		
Male	45	47.9		
Age				
Visit 1	94		9.80	3.74
Visit 2	76		14.24	3.86
Visit 3	56		18.06	4.47
Racialized identity				
Black British	94	100		
Index of Multiple Deprivation				
1 (most deprived)	35	37.2		
2	26	27.6		
3	17	18.1		
4	15	16.0		
5 (least deprived)	1	1.1		
Genotype				
HbSS	93	99		
HbSβ0	1	1		
Prescribed hydroxyurea				
Yes	7	14.9		
No	84	82.0		
Not reported	3	3.1		
Chronic transfusion				
Yes	9	10.9		
No	86	89.1		
Asthma diagnosis				
Yes	8	8.6		
No	86	91.4		
Hemoglobin			87.3	15.03
Hematocrit			25.8	4.71

*Note.* Other than age, all characteristics reported were collected at baseline.

Table 2 demonstrates that the mean composite score for CSHQ, our measure of sleep quality, was 13.91. Ten percent of caregivers reported poor sleep quality for children (i.e., scores above 30, the clinical cutoff). CYA averaged 7.94 pain-related hospitalizations since birth and 0.81 between study visits. They averaged 1.38 ACS-related hospitalizations since birth and 0.12 between study visits. A one-sample *t*-test indicated that NO_2_ levels across Visits 1 and 2 were significantly higher than the UK Air Quality Standards Regulations recommended mean concentration levels, whereas PM_10_ were significantly lower and well within recommended levels (see Table 3). CYA completed cognitive testing at Visit 3. The WMI, PSI, and D-KEFS Tower subtest scores were available for 56 participants, while the caregiver-completed BRIEF questionnaire was available for 37 participants. Twenty-one percent of the CYA had clinically elevated scores for BRIEF GEC (i.e., > 65).

**Table 2.** Sleep quality scores and pain and ACS-related hospitalizations.

Measures	No. of participants	*M* (*SD*)
Visit 2 Child Sleep Habit Questionnaire total score	56	13.91 (1.31)
Yearly rate of pain-related hospitalizations since birth	94	7.94 (7.41)
Yearly rate of pain-related hospitalizations between Visits 1 and 2	56	0.81 (0.11)
Yearly rate of ACS-related hospitalizations since birth	94	1.38 (1.95)
Yearly rate of ACS-related hospitalizations between Visits 1 and 2	56	0.12 (0.02)

*Note.* ACS = acute chest syndrome.

**Table 3.** Air pollution measures between Visits 1 and 2 and Visits 1 and 3 and differences between obtained and expected (UK Air Standards) values.

Pollutant	Sample mean	Annual average	Mean difference	Difference between the sample and the annual average values		
				*t*	*p*	Cohen’s *d*
	*M* (*SD*)					
Nitrogen dioxide Visit 1–2 (*N* = 63)	52.4 (31.19)	40	12.47	3.17	.002	.400
Nitrogen dioxide Visit 1–3 (*N* = 50)	48.5 (26.33)	40	8.50	2.28	.027	.323
Particulate matter >10 (PM_10_) Visit 1–2 (*N* = 55)	25.65 (4.84)	40	−14.35	−24.18	<.001	−2.95
Particulate matter >10 (PM_10_) Visit 1–3 (*N* = 42)	24.27 (4.21)	40	−15.72		<.001	−3.73

**Table 4.** Regression analyses assessing the influence of air pollutants on pain and ACS-related hospitalizations controlling for age, asthma diagnosis, and the Index of Multiple Deprivation.

Dependent variable: lifetime pain-related hospitalizations Overall model: *N* = 47, *R*^2^ = .21, sig. = .068						
Effect	*B*	*SE*	β	95% CI		*p*
Age at Visit 2	.65	.27	.34	.09	1.2	.02
Asthma Diagnosis	1.7	3.7	.07	−5.8	9.2	.65
IMD	−3.3	2.2	−.22	−7.8	1.2	.15
Nitrogen dioxide Visits 1–2	.07	.06	.32	−.04	.18	.21
Particulate matter >10 (PM_10_) Visits 1–2	−.55	.38	−.36	−1.3	.22	.16

Dependent variable: lifetime ACS-related hospitalizations Overall model: *N* = 47, *R*^2^ = .31, sig. = .005						
Effect	*B*	*SE*	β	95% CI		*p*
Age at Visit 2	.02	.07	.04	−.12	.17	.75
Asthma diagnosis	2.5	.99	.35	.47	4.5	.02
IMD	−1.1	.60	−.25	−2.3	.11	.07
Nitrogen dioxide Visit 1–2	.04	.02	.62	.01	.07	.01
Particulate matter >10 (PM_10_) Visits 1–2	.29	.10	.65	.50	.08	.007

Dependent variable: ACS-related hospitalizations between Visits 1 and 2 Overall model: *N* = 48, *R*^2^ = .23, sig. = .038)						
Effect	*B*	*SE*	β	95% CI		*p*
Age at Visit 2	−.01	.01	−.14	−.02	.01	.32
Asthma Diagnosis	.22	.08	.40	.06	.39	.01
IMD	−.04	.05	−.12	−.15	.06	.41
Nitrogen dioxide Visits 1–2	.001	.001	.24	−.001	.004	.32
Particulate matter >10 (PM_10_) Visits 1–2	.01	.01	.26	.03	.01	.30

*Note.* Nitrogen dioxide and PM_10_ are measured in µg/m^3^. CI = confidence interval; ACS = acute chest syndrome; IMD = Index of Multiple Deprivation.

### Regression analyses

Our first logistic regression indicated that neither air pollutants (NOx and PM_10_) significantly predicted whether CYA lived in a more or less deprived area (*p* = .28). The second logistic regression indicated that air pollutants (NOx and PM_10_) trended toward predicting whether CYA had an asthma diagnosis, χ^2^(2, *N* = 53) = 5.25, *p* = .07. The model explained 19% of the variance in asthma diagnosis and correctly classified 88.7% of cases. NO_2_ (*OR* = 1.055, *p* = .06) and PM_10_ (*OR* = .63, *p* = .06) concentrations predicted an increased likelihood of asthma diagnosis.

Multiple linear regression analyses indicated that air pollutants did not significantly predict sleep quality, *R*^2^ = .01, *F*(5, 46) = 0.13, *p* = .99, or pain-related hospitalizations between Visits 1 and 2, *R*^2^ = .15, *F*(5, 43) = 1.51, *p* = .21. However, lifetime pain-related hospitalizations trended toward significance, with older age as the only significant independent predictor. The overall model for lifetime ACS-related hospitalizations was significant, with the control variables of asthma diagnosis and IMD significant and trending toward significance, respectively. Additionally, both air pollutants were significant independent predictors. The overall model for ACS-related hospitalizations between Visits 1 and 2 was significant, but the only significant predictor was the control variable of asthma diagnosis (see Table 4).

Multiple linear regression models were also conducted to determine if, after controlling for IMD, air pollutants predicted executive function across WMI, PSI, the Tower subtest, as well as the caregiver-reported BRIEF. However, none of the models were significant.

## Discussion

Environmental pollution remains high in densely populated cities where concentrations of air pollutants are typically elevated due to vehicular emissions and industrial activities (Fairburn et al., 2019). By leveraging data from the London Air Quality Network and aligning it with participants’ residential locations, our preliminary study was a secondary analysis that examined the role of these pollutants on CYA living with SCD in London, United Kingdom, a population inherently vulnerable due to the pathophysiological characteristics of SCD and socio-environmental factors. Specifically, we investigated the role of environmental exposure to NO_2_ and PM_10_, primarily focusing on how these pollutants influenced pain and ACS-related hospitalizations, sleep quality, executive function, and processing speed. Our findings revealed that air pollution, particularly NO_2_, was significantly associated with lifetime ACS-related hospitalizations, and community deprivation played a role in this outcome. The presence of asthma further increased the likelihood of lifetime ACS-related hospitalizations, underscoring the compounded risk that asthma poses for CYA living with SCD. As ACS is a severe complication of SCD, this result indicates that environmental pollutants may exacerbate respiratory complications in CYA living with SCD (Blumberg et al., 2020).

Conversely, the effects of air pollution on pain-related hospitalizations, sleep quality, and executive function were less pronounced, though some trends toward significance were observed. However, these results contrast with previous studies that have linked air pollution to various health complications in children, including sleep disturbances and cognitive impairments. This is noteworthy given the established evidence suggesting that exposure to fine PM_2.5_ can penetrate the blood–brain barrier, leading to neuroinflammation and cognitive deficits (Block & Calderón-Garcidueñas, 2009; Calderón-Garcidueñas et al., 2011). The lack of a significant relationship between air pollutants and cognitive outcomes in this study may be attributed to several factors. First, the measurement of cognitive function was focused on specific domains (working memory, processing speed, and executive function), as these are the domains in which CYA living with SCD typically experience difficulties (Prussien et al., 2019). It is also possible that environmental exposures might potentially influence other cognitive domains (e.g., verbal and perceptual reasoning). Another consideration is the possibility of a delayed cognitive impact, where cognitive deficits related to air pollution exposure may manifest later in life beyond the study’s follow-up period. Further, although relevant to respiratory health, the specific pollutants examined—NO_2_ and PM_10_—may have played a different role in cognitive outcomes compared to finer PM like PM_2.5_.

Our data underscore the need for further research into the health impacts of air pollution on marginalized populations, particularly those with chronic conditions like SCD. Future research could expand the scope to include a more diverse range of pollutants, such as PM_2.5_ and carbon monoxide, which could provide a more comprehensive picture of environmental risks. As a next step, it would be important to explore the potential mechanisms through which air pollution may contribute to ACS-related hospitalizations as early life exposure to air pollutants was significantly associated. Understanding the biological pathways involved, such as systemic inflammation and oxidative stress, could help clarify the link between environmental exposures and respiratory complications in this population and offer valuable insights for targeted interventions (Arias-Pérez et al., 2020). For example, strategies to mitigate the adverse effects of air pollution, such as access to green spaces, air purification systems, and air quality improvements, might mitigate some of the adverse effects observed in our study. Finally, qualitative studies exploring the lived experiences of CYA living with SCD in polluted environments could complement quantitative findings, offering a more holistic understanding of how environmental stressors impact daily functioning and quality of life (Khan et al., 2023).

Using longitudinal data from the London Sleep Asthma Cohort, we assessed long-term trends in exposure and outcomes, providing a greater understanding of how environmental factors affect SCD outcomes. The use of multiple indicators, including hospitalizations, sleep quality, and cognitive assessments, allowed for a multifaceted evaluation of the effects of air pollution. Focusing on a predominantly Black chronic illness population living in London is essential, given the intersection of racialized identity, community deprivation, and environmental health disparities. Despite these strengths, there are limitations to our study that warrant discussion. First, the reliance on air quality data from monitoring stations rather than personal exposure data may not accurately reflect the individual exposure levels experienced by participants (Kortoçi et al., 2022), particularly given variations in time spent indoors versus outdoors. This limitation is especially relevant in SCD, where time spent outdoors may vary widely. Second, although sufficient for certain analyses, the sample size may have limited our ability to detect smaller effects, especially concerning cognitive function and sleep quality. Third, the study’s observational nature precludes the establishment of causality between air pollution exposure and health outcomes. Fourth, our study included some participants receiving disease-modifying therapies. We did not exclude these participants from our analyses as they had begun use just before or during Visit 1. However, we cannot discount that their inclusion may have influenced our findings as these therapies generally improve underlying disease pathology. Lastly, the transferability of our findings may be limited, as the cohort was drawn from densely populated areas in London, and results may not apply to rural settings or other cities with different pollution profiles.

The findings of this study have significant implications for public health policies, clinical practices, and community interventions aimed at improving the health outcomes of CYA living with SCD. The association between air pollutants and increased ACS-related hospitalizations underscores the urgent need for environmental interventions to reduce exposure levels in vulnerable populations. The implementation of the ultra-low emission zone (ULEZ) in London (i.e., where an emissions standard-based charge is applied to non-compliant road vehicles) (Ma et al., 2021) represents a pivotal step toward mitigating air pollution by restricting high-emission vehicles in designated areas, thereby lowering concentrations of NO_2_ and PM_10_ (Bishop and Bornioli, 2022). The ULEZ has the potential to significantly improve air quality, particularly in areas with high concentrations of marginalized communities disproportionately affected by pollution (Ma et al., 2021). Indoor air pollutants also pose a health risk. They can include PM sources such as smoking, cooking, heating, candles, and insecticides, whereas volatile organic compounds include cleaning materials, glue, and personal care products. Studies of indoor pollution in the general population have shown associations between PM and lung function, reduced oxygen saturation, and increased risk of asthma (Du et al., 2025). Given the nature of SCD and factoring in economic deprivation, a potential research avenue is the use of low-cost air quality monitors to assess individual exposure, providing a more accurate picture of the participants’ exposure levels and potential health outcomes (Wang et al., 2020).

From a policy perspective, the study highlights the intersection of environmental health and social justice. Black communities in the United Kingdom, already facing socioeconomic disadvantages, are further burdened by higher levels of environmental pollution. Policymakers must prioritize environmental justice by ensuring equitable resource distribution and interventions to reduce pollution in the most affected areas. This includes expanding initiatives like ULEZ, enhancing green infrastructure, and enforcing stricter emissions regulations to protect minoritized populations (Hewitt et al., 2020). From a clinical perspective, pediatric psychologists could add the risk of indoor and outdoor exposure to pollution to their screening tools when collecting patient history, share resources with families (e.g., websites), and provide practical advice such as using air purifiers when possible and minimizing outdoor activities on higher pollution days (Hadley et al., 2018).

## Conclusion

Our preliminary study provides important insights into the complex relationship between environmental pollutants and health outcomes in CYA living with SCD in London. Although air pollution was not found to be significantly associated with cognitive outcomes or sleep quality, it was associated with an increased risk of ACS-related hospitalizations, particularly among those with asthma. These findings underscore the need for continued research into the effects of air pollution on this minoritized population, as well as targeted interventions to reduce exposure and improve health outcomes. Addressing the environmental and social determinants of health for children with SCD will be critical in reducing health disparities and promoting equity in care.
